# Investigation on Spectrum-Effect Correlation between Constituents Absorbed into Blood and Bioactivities of Baizhu Shaoyao San before and after Processing on Ulcerative Colitis Rats by UHPLC/Q-TOF-MS/MS Coupled with Gray Correlation Analysis

**DOI:** 10.3390/molecules24050940

**Published:** 2019-03-07

**Authors:** Hao Cai, Yangyang Xu, Li Xie, Yu Duan, Jia Zhou, Jing Liu, Minjie Niu, Yating Zhang, Lin Shen, Ke Pei, Gang Cao

**Affiliations:** 1 School of Pharmacy, Nanjing University of Chinese Medicine, Nanjing 210023, China; yangyangxu92@126.com (Y.X.); xl20160806@126.com (L.X.); duanyu1681@sina.com (Y.D.); zhoujia19931005@126.com (J.Z.); 15951921665@163.com (J.L.); someonearis@163.com (M.N.); zhangyatingzyt@126.com (Y.Z.); sonesunfany@sina.cn (L.S.); 2 Engineering Center of State Ministry of Education for Standardization of Chinese Medicine Processing, Nanjing University of Chinese Medicine, Nanjing 210023, China; 3 Institute of Pharmaceutical and Food Engineering, Shanxi University of Traditional Chinese Medicine, Taiyuan 030024, China; peike_pk@126.com; 4 School of Pharmacy, Zhejiang Chinese Medical University, Hangzhou 310053, China

**Keywords:** Baizhu Shaoyao San, spectrum-effect correlation, UHPLC/Q-TOF-MS/MS, ulcerative colitis, gray correlation analysis

## Abstract

Baizhu Shaoyao San (BSS) is a crucial traditional Chinese medicinal formula widely applied for the treatment of painful diarrhea, diarrhea-predominant irritable bowel syndrome, ulcerative colitis, and some other gastrointestinal diseases. Corresponding to the clinical medication, the three medicinal herbs (Atractylodis Macrocephalae Rhizoma, Paeoniae Radix Alba, and Citri Reticulatae Pericarpium) included in BSS should be processed using some specific methods of stir-frying. To find the underlying correlations between serum chemical profiles and curative effects of crude and processed BSS on ulcerative colitis rats, and further explore for the effective material basis of processing, an UHPLC/Q-TOF-MS/MS technique coupled with gray correlation analysis (GCA) was developed. A total of 134 compounds were identified in rat sera after oral administration of BSS, among which 24 compounds were prototypes and 110 compounds were metabolites. Meanwhile, an ulcerative colitis model was established in rats by enema with 2,4,6-trinitrobenzene sulfonic acid, and the pharmacodynamic indicators for drug efficacies were evaluated as well. According to the results, processed BSS showed better efficacy than crude BSS. The top 10 potential effective components with high degree of correlation were identified based on GCA results, which were thought to be the crucial compounds that contributed to the enhancement of therapeutic effects in BSS after processing.

## 1. Introduction

As a famous and classic herbal formula in traditional Chinese medicine (TCM), Baizhu Shaoyao San (BSS) is composed of Atractylodis Macrocephalae Rhizoma (Asteraceae), Paeoniae Radix Alba (Ranunculaceae), Citri Reticulatae Pericarpium (Rutaceae), and Saposhnikoviae Radix (Umbelliferae) at a ratio of 6:4:3:4. It was first recorded in “Dan-Xi-Xin-Fa”, a comprehensive medical treatise edited by distinguished physician Zhenheng Zhu (1281–1358 A.D.) in the Yuan Dynasty, to be commonly used as a mediative formula for softening liver and tonifying spleen, as well as eliminating dampness and alleviating diarrhea [1]. Nowadays, BSS is commonly used in the clinic for various gastrointestinal-related diseases, such as painful diarrhea, ulcerative colitis, and diarrhea-predominant irritable bowel syndrome [2,3]. Actually, the efficacies of BSS are the synergistic results of some bioactive substances from each medicinal herb contained in the formula, such as sesquiterpene lactones in Atractylodis Macrocephalae Rhizoma, monoterpene glycosides in Paeoniae Radix Alba, flavonoids and flavonoid glycosides in Citri Reticulatae Pericarpium, and coumarins and chromones in Saposhnikoviae Radix, which reveal the so-called prescription compatibility (fufang peiwu) characteristics of TCM [4,5]. In addition to TCM prescription compatibility, TCM processing is another feature according to the theory of TCM. Together they constitute the two indispensable factors that distinguish TCM from natural medicine. As an ancient pharmaceutical technology, a variety of processing methods are employed to process the crude medicinal herbs, including washing, cutting, baking, frying, calcining, roasting, steaming, blanching, and others, exerting the significant effects of reducing toxicity, relieving drug irritation, enhancing therapeutic effect, and increasing clinical applicability [6,7,8]. Current studies are mainly focused on the TCM processing effect on individual herbs, and few researchers have examined the effects of processing on Chinese herbal formulas, which could result in inconsistency with clinical medication practice [9,10]. On the basis of the theory of TCM and the Chinese Pharmacopoeia, the three herbs contained in BSS should be processed with specific traditional techniques to decrease harmful side-effects and improve the curative efficacy of the whole prescription. To be specific, Atractylodis Macrocephalae Rhizoma should be stir-fried using honey-processing wheat bran, Paeoniae Radix Alba should be stir-fried using wheat bran, and Citri Reticulatae Pericarpium should be stir-fried alone without any auxiliary materials. According to some recent reports [11,12], significant changes of therapeutic effects in these three medicinal herbs have been observed after the processing procedures. However, how the processing of medicinal herbs contained in BSS influences the therapeutic effect of the whole medicinal formula has not been investigated, causing inconsistencies with the clinical medication. Therefore, it is of great necessary and significance to elucidate the difference on curative effects of BSS before and after processing and make clear what the effective material basis is hidden behind the changes.

Due to the extreme complexity of the ingredients in TCM, not all components play equally important roles in the treatment of diseases. The clinical efficacy of TCM is the synergistic effect of various active compounds, and identifying these effective substances is an essential step for better elucidating the functionary mechanism and processing principle of TCM by modern science. In our previous study, it was found that significant changes of both volatile and non-volatile components in BSS had taken place after processing [13,14]. According to the serum pharmacochemistry theory of TCM, only the components absorbed into the blood could exert their therapeutic effects [15]. Thus, in this paper, the methodology of serum pharmacochemistry was used to investigate the fingerprint chromatograms of rat sera after oral administration of BSS, and search for the prototypes and metabolites of the constituents of BSS in rat sera.

Because of the numerous components in TCM herbal formulae and their complicated metabolic pathways, ultra-high-performance liquid chromatography (UHPLC) coupled with quadrupole, hybrid orthogonal acceleration time-of-flight tandem mass spectrometry (Q-TOF-MS/MS) has been increasingly employed as an important analytical method due to its high resolution, good sensitivity, and precise mass value of both precursor and product ions [16,17]. In addition, UHPLC/Q-TOF-MS/MS is also a powerful tool for identifying unknown compounds without reference substances in vitro or in vivo. Therefore, in this paper, it was employed for comprehensively qualitative and semi-quantitative analysis of the prototypes and metabolites in rat sera after oral administration of crude and processed BSS.

The spectrum-effect correlation is a modern research approach of TCM, which clarifies the relationship between chemical fingerprints and bio-effects in a series of samples using chemometric and statistical analysis techniques [18,19]. Gray correlation analysis (GCA) is a kind of correlation analysis method and generally employed to indicate the quantitative comparison of the development trend in a dynamic variation system. 

Compared with other statistical methods including cluster analysis and canonical correlation analysis, GCA possesses the advantages of requiring small sample size and computational amount, which has been applied widely [20]. In this paper, GCA was employed to investigate the relationship between constituents absorbed into blood and biological activities of crude and processed BSS in ulcerative colitis rats at different time-points during the treatment process. Based on this method, it could reveal the efficacy-related components and uncover the effective material basis of TCM processing in BSS.

## 2. Results and Discussion

### 2.1. UHPLC/Q-TOF-MS/MS Analysis of Constituents Absorbed into Blood

The typical total ion chromatograms (TICs) of compounds in rat sera after 3 days of administration of crude and processed BSS in negative and positive ion modes are shown in Supplementary Figure S1. The MetabolitePilot^TM^ and PeakView^TM^ softwares were used to extract and identify the constituents absorbed into blood. By comparing with the chromatogram of control serum sample, 134 peaks were identified in drug-containing serums, which consisted of 24 prototype components in BSS and 110 metabolites. The detailed information of the identification results and MS data are shown in Table 1. It could be concluded from the results that the metabolic pathways of lactones from Atractylodis Macrocephalae Rhizoma in rats mainly include glucuronidation, hydroxylation, hydrolysis, and hydrogenation, and reciprocal transformation is possible; the metabolic pathways of glycosides from Paeoniae Radix Alba mainly include methylation, deglycosylation, benzoyloxy group loss, glycine conjugation, and glutamine conjugation; the metabolic pathways of flavonoids from Citri Reticulatae Pericarpium mainly include demethylation, demethoxylation, hydroxylation, methylation, glucuronidation, and deglycosylation; the metabolic pathways of chromones from Saposhnikoviae Radix mainly include deglycosylation, demethoxylation, hydroxylation, and deacetylation; the metabolic pathways of Coumarins from Saposhnikoviae Radix mainly include methylation, hydroxylation, and isomerization.

**Figure 1:**
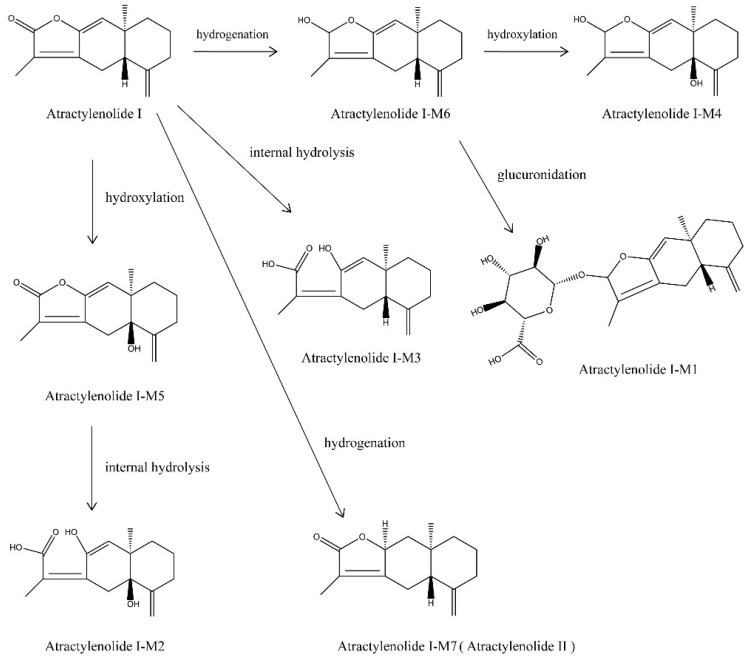
Proposed metabolic pathways of atractylenolide I in rat sera.

As a typical example, the proposed metabolic pathways of atractylenolide I in rat sera are illustrated in Figure 1.

### 2.2. Results of Anti-Ulcerative Colitis Effects

#### 2.2.1. Effects of BSS on Serum Cytokines before and after Processing

**Figure 2:**
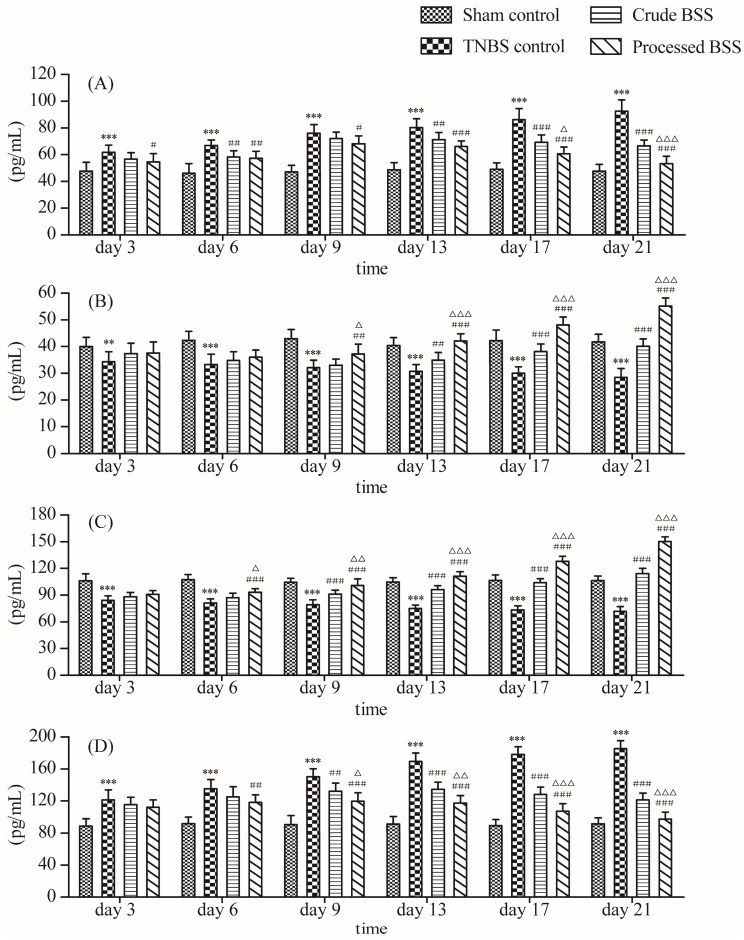
Results of IL-6, IL-10, TGF-β1, and TNF-α levels in rat serums after consecutive administration of crude and processed BSS on day 3, 6, 9, 13, 17, and 21 (*n* = 10). (**A**): serum IL-6 level; (**B**): serum IL-10 level; (**C**): serum TGF-β1 level; (**D**): serum TNF-α level. ** *p* < 0.01, *** *p* < 0.001 vs. sham control; ^#^ *p* < 0.05, ^##^ *p* < 0.01, ^###^ *p* < 0.001 vs. TNBS control; ^Δ^ *p* < 0.05, ^ΔΔ^ *p* < 0.01, ^ΔΔΔ^ *p* < 0.01 vs. crude BSS.

The results of IL-6, IL-10, TGF-β1, and TNF-α levels in rat sera after consecutive administration of crude and processed BSS on day 3, 6, 9, 13, 17, and 21 are shown in Figure 2.

The results showed that the levels of TNF-α, IL-6, IL-10, and TGF-β1 in rat sera of the 2,4,6-trinitrobenzenesulfonic acid (TNBS) control group were significantly different from those of the sham control group, indicating that the model of ulcerative colitis was successfully established. Specifically, compared with the sham control group on day 3, 6, 9, 13, 17, and 21, the levels of IL-6 and TNF-α in rat serums were significantly elevated (*p* < 0.001) and the levels of IL-10 and TGF-β1 in rat serums were significantly decreased (*p* < 0.01), indicating that the rats developed extensive inflammatory responses. However, compared with the TNBS control group, all serum cytokine levels in crude and processed BSS groups were improved to some extents. Especially after drug administration on day 13, the BSS groups exhibited significant difference on cytokine levels from the TNBS control group, among which the processed BSS group led to a better efficacy than the crude BSS group. The results proved that BSS could inhibit the development of ulcerative colitis by reducing the expression of pro-inflammatory cytokines and increasing the expression of anti-inflammatory cytokines in serums, which verified the theory that processing can increase curative effects.

#### 2.2.2. Effects of BSS on Disease Activity Index (DAI) before and after Processing

After one day of modeling, the TNBS-treated rats showed positive reaction in hemoccult test and displayed varying degrees of loose stool. Mental slackness, lazy eating, weight loss, vertical hair, and arched back gradually appeared in the TNBS control group throughout the experiment process. After about seven days of treatment, the conditions of activity, diarrhea, blood stool, and weight loss in the crude BSS, processed BSS, and salazosulfapyridine (SASP) groups were all alleviated to some extent. Table 2 shows the DAI score results after 20 days of drug administration. Compared with the sham control group, DAI was significantly higher in the TNBS control group (*p* < 0.001). Treatments with SASP, crude, and processed BSS significantly prevented TNBS colitis-induced weight loss and DAI increasement (*p* < 0.001). Besides, DAI was markedly lower in the processed BSS group than the crude processed BSS group (*p* < 0.001).

#### 2.2.3. Effects of BSS on Macroscopic and Histopathological Alterations before and after Processing

Compared with the sham control group, the macroscopic colon damage scores of the TNBS control group were markedly increased (*p* < 0.001). However, the damage scores showed a significant decrease in the crude BSS, processed BSS, and SASP groups (*p* < 0.001), among which the processed BSS group exhibited better efficacy than the crude BSS group (*p* < 0.01). The results are shown in Table 2.

**Figure 3:**
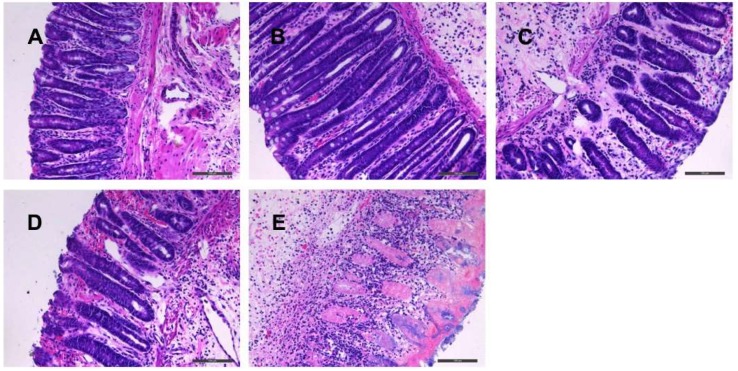
Histopathological results of colon tissues in rats from different groups (HE, × 200). (**A**): sham control group; (**B**): SASP group; (**C**): processed BSS group; (**D**): crude BSS group; (**E**): TNBS control group.

The hematoxylin-eosin (HE) staining of pathological sections of rat colon tissues is shown in Figure 3, where the obvious histopathological differences of each group can be observed. The colon tissue structures from the sham control group were complete and continuous, the glands arranged regularly with clear structures, and there were no inflammatory cell infiltration or ulceration. The colon tissues from the TNBS control group exhibited hyperemia, vascular hyperplasia, edema, ulcer formation, massive inflammatory cell infiltration, and partial mucosal necrosis. Intestinal glands and crypts were also disappeared. Compared with the TNBS control group, colonic tissues of rats administered with crude BSS, processed BSS, and SASP were all recovered to some extent. The colon structures of the SASP group were generally clear and intact, the glands were well arranged again, with a small amount of inflammatory cell infiltration and mucosal congestion. An improved colon structure was observed in the crude BSS group, the inflammatory cell infiltration and vascular hyperplasia were mild, but still accompanied with moderate congestion of mucosa. Compared with crude BSS, the processed BSS group was demonstrated better performance in repairing colon injury, the tissue structures were relatively complete and continuous with significant reduction of inflammatory cell infiltration and mucosal congestion.

#### 2.2.4. Effects of BSS on Protein Expression in TLR4 Signaling Pathway before and after Processing

**Figure 4:**
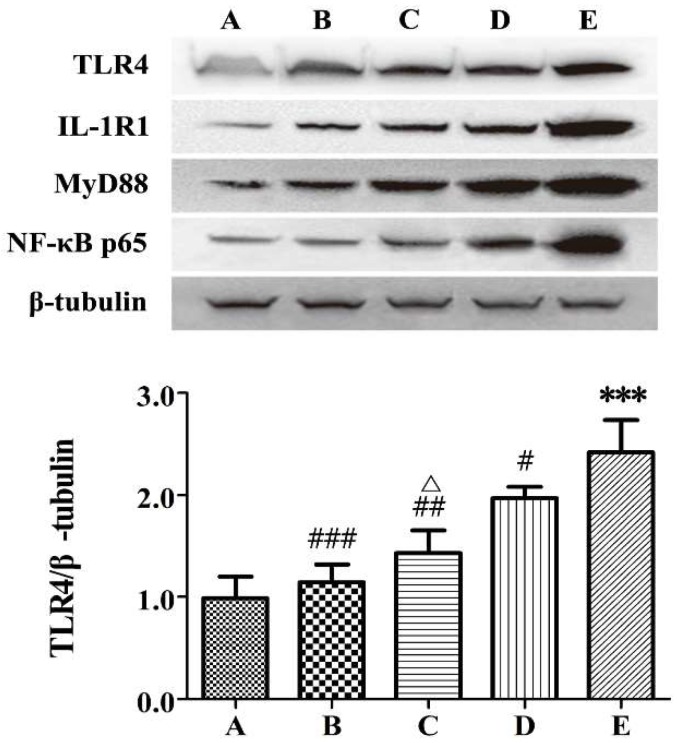

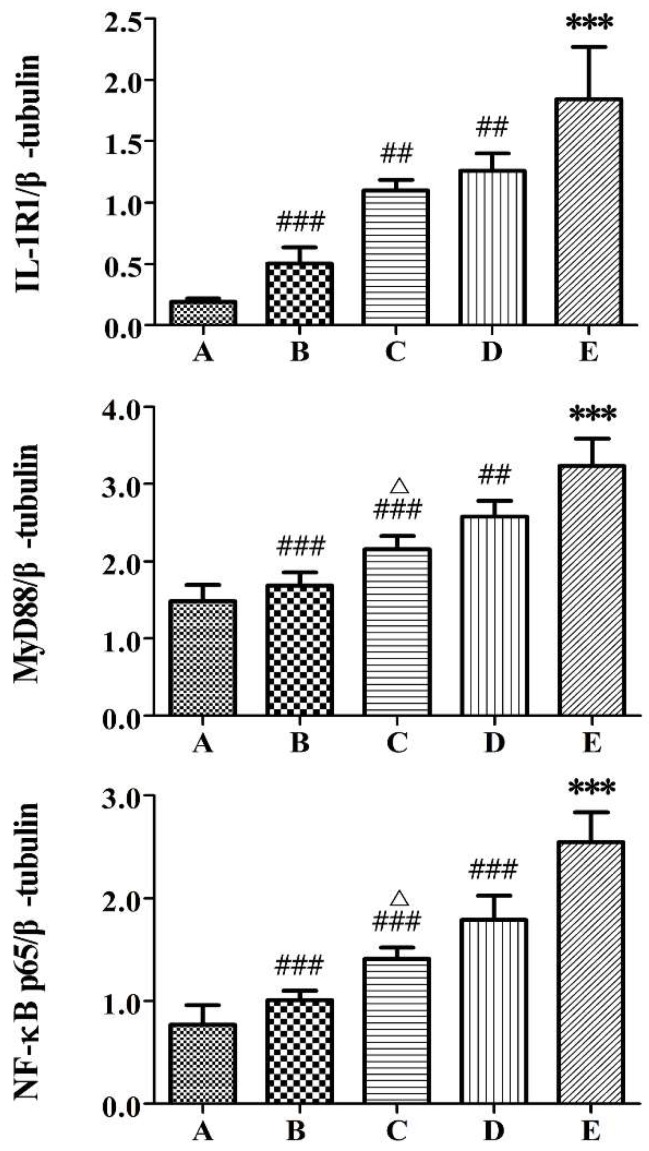
Protein expressions of TLR4, IL-1R1, MyD88, and NF-κB p65 in rat colons (*n* = 6). A: sham control group; B: SASP group; C: processed BSS group; D: crude BSS group; E: TNBS control group. *** *p* < 0.001 vs. sham control; ^#^ *p* < 0.05, ^##^ *p* < 0.01, ^###^ *p* < 0.001 vs. TNBS control; ^Δ^ *p* < 0.05 vs. crude BSS.

The influence of BSS on the activation of TLR4 signaling pathway was examined. We assessed a series of signaling molecules, including TLR4, IL-1R1, MyD88, and NF-κB p65 by western Blot, and the results are shown in Figure 4.

As can be seen from the figure, the TLR4, IL-1R1, MyD88, and NF-κB p65 protein expression in the inflamed colon was significantly increased in the TNBS control group compared with the sham control group (*p* < 0.001). Treatment with Crude BSS, processed BSS, and SASP markedly inhibited TNBS-induced increase of these TLR4-relevant protein expressions (*p* < 0.05). Compared with crude BSS, processed BSS exhibited a stronger ability in regulating the high expression of TLR4, MyD88, and NF-κB p65 (*p* < 0.05). Processed BSS also decreased the IL-1R1 level more in the inflamed colon compared with crude BSS, but the difference was not significant.

### 2.3. Results of Gray Correlation Analysis

The calculated results of the inhibition and promotion rates of the cytokine levels after administration of crude and processed BSS on day 3, 6, 9, 13, 17, and 21 are shown in Table 3. GCA was used for investigating the spectrum–effect relationships between the area values of 134 compound peaks detected by UHPLC fingerprints and the anti-ulcerative colitis efficiency characterized by the inhibition and promotion rates of inflammatory cytokines at different time points. Then, the gray correlation degree between these two sets of parameters were calculated, in which the top 10 potential effective components associated with each cytokine sorted according to the degree of correlation are shown in Table 4.

According to the gray correlation grades listed in Table 4, for each different pharmacodynamic index, the constituents absorbed into blood with relatively high correlation were also different. Among these correlative constituents, it was found that atractylenolide I-M5, a hydroxylated metabolite of atractylode I, was highly correlated with all inflammatory cytokines including TNF-α, IL-6, IL-10, and TGF-β1. In addition, atractylenolide II, atractylenolide III, hydrogenated metabolite of atractylenolide III (atractylenolide III-M5), two hydroxylated metabolites of atractylenolide III (atractylenolide III-M3 and atractylenolide III-M4), hydroxylated and hydrolyzed metabolite of atractylenolide I (atractylenolide I-M2), and di-hydroxylated metabolite of atractylenolide II (atractylenolide II-M2) also demonstrated strong correlation with anti-colitis effect. These atractylenolide-related compounds were all belong to the main ingredients of Atractylodis Macrocephalae Rhizoma (the monarch drug of BSS), and exhibited extensive pharmacological activities including protecting intestinal mucosa, inhibiting inflammation, regulating gastro- intestinal peristalsis, and boosting immunity, according to numerous relative literature [21,22,23,24]. At the same time, based on our previous study [14], the contents of atractylenolide I, atractylenolide II, and atractylenolide III in BSS were significantly increased after processing, and thus the increasement of these components eventually led to the enhancement of in vivo effects, which validated the ancient theory of TCM that ‘processing enhancing efficacy’. Meanwhile, it could be interestingly found that most of the metabolites possessed higher grades of correlation in treating colitis compared with the prototype compounds. Through some recent literature [25,26], the lack of hydrophilic groups in the chemical structures of atractylenolide I and atractylenolide II resulted in their low bioavailability after oral administration, while atractylenolide III contained a hydrophilicity structure of hydroxyl, which greatly increased its absorption into blood. Similarly, these atractylenolide compounds increased water solubility through in vivo metabolisms, and thus increased the ratio of blood absorption and enhanced the bioavailability, which could be partially explained that why the metabolites of atractylenolides owned relative higher relevance with the treatment of ulcerative colitis and suggested that the drug metabolism of BSS played a vital role during the process of treatment.

In addition to Atractylodis Macrocephalae Rhizoma (monarch drug), a lot of constituents and their metabolites of Paeoniae Radix Alba (minister drug), Citri Reticulatae Pericarpium (assistant drug), and Saposhnikoviae Radix (guide drug) also exhibited high correlations with the therapeutic effects. These compounds consisted of albiflorin-M1 and albiflorin-M2 from Paeoniae Radix Alba, hesperidin-M2, nobiletin-M1, nobiletin-M3, 3,5,6,7,8,3′,4′-heptamethoxyflavone-M9, sinensetin-M1, 3,5,7,3′,4′-pentamethoxyflavone-M2, dimethyl anthranilate-M1, and tangeretin-M4 from Citri Reticulatae Pericarpium, and cimifugin, cimifugin-M1, cimifugin-M3, 5-*O*-methylvisamminol-M2, sec-*O*-glucosylhamaudol, prim-*O*-glucosylcimifugin, and D-Mannose from Saposhnikoviae Radix. Meanwhile, it was reported that albiflorin, hesperidin, nobiletin, tangeretin, and prim-*O*-glucosylcimifugin have the effects of protecting intestinal barrier and resisting experimental colitis [27,28,29,30,31], which were definitely in accordance with the results in our study. According to our previous study [14], among the compounds above, only the content of albiflorin in BSS significantly increased after processing, while other compounds did not change significantly before and after processing, nevertheless, all these components were positively correlated with the enhancement of efficacy after processing.

There may be two reasons for this interesting phenomenon. The first reason could be inferred that the intestinal microbial compositions and hepatic drug metabolic enzymes in vivo are too much complicated, and thus some structurally similar constituents with increased contents after processing could be transformed into the above-mentioned metabolites through a series of complex metabolic procedures in the body. For example, catechin, luteolin-7-*O*-rutinoside, 6-methoxy- naringenin, and nobiletin isomer are flavonoids with increased contents in BSS after processing based on a previous study [14], they share the same parent structure with the flavonoid metabolites that correlated with efficacy enhancement, and probably could be transformed into the target metabolites through some unknown pathways. The second reason might be related to the drug interaction effects in vivo. Although some components in BSS might not exert therapeutic effects by themselves, the variation of contents after processing might influence the absorption and metabolism of other components by affecting the binding sites of membrane transport proteins and the biological activities of drug-metabolizing enzymes, which was closely related to the compatibility theory of TCM according to some published literature [32,33,34,35].

Processing and compatibility together constitute the characteristics of clinical medication in TCM, and that is what TCM can be distinguished from absolute natural medicine [36,37]. As a splendid treasure of science and culture in China, TCM embodies profound and sapiential wisdom and holistic and systematic health concepts. The results of our study provided supporting evidence for the theory concerning the synergistic effects of processing and compatibility in TCM systems. Additionally, the GCA results demonstrated the material basis of BSS, which could be helpful for deeper exploration on its mechanism of action. Also, it would be considered as a primary evaluation standard of BSS for quality control during its clinical application.

## 3. Materials and Methods

### 3.1. Materials and Reagents

Medicinal herbs of Crude Atractylodis Macrocephalae Rhizoma, Paeoniae Radix Alba, Citri Reticulatae Pericarpium, and Saposhnikoviae Radix were all obtained from Jingwan TCM Pieces Factory (Bozhou, China), and were authenticated by Professor Hao Cai, who is the expert in the TCM field. Processed samples of Atractylodis Macrocephalae Rhizoma, Paeoniae Radix Alba, and Citri Reticulatae Pericarpium were prepared corresponding to the processing specifications in the Chinese Pharmacopoeia. Voucher specimens of all medicinal herbs were deposited in School of Pharmacy, Nanjing University of Chinese Medicine (Nanjing, China).

Methanol (LC/MS grade) and acetonitrile (LC/MS grade) were purchased from E. Merck (Darmstadt, Germany). Purified water was acquired from a Milli-Q purification system (Millipore, Bedford, MA, USA). Ethyl acetate (HPLC grade) was purchased from Sinopharm Chemical Reagent Co., Ltd. (Shanghai, China). 2,4,6-Trinitrobenzene sulfonic acid (TNBS) aqueous solution (5%) was purchased from Sigma-Aldrich (Oakville, ON, Canada). Salazosulfapyridine (SASP) was purchased from Shanghai Fuda Pharmaceutical Co., Ltd. (Shanghai, China). 4% paraformaldehyde was purchased from Biosharp (Beijing, China). Stool occult blood test kit was purchased from Jian Cheng Biological Technology Co., Ltd. (Nanjing, China). Antibodies of TLR4 (sc-293072), MyD88 (sc-74532), NF-κB p65 (sc-8008), and IL-1R1 (sc-393998) were purchased from Santa Cruz Biotechnology, Inc. (Santa Cruz, CA, USA). ELISA kits of TNF-α, IL-6, IL-10, and TGF-β1 were purchased from Nanjing Jin Yibai Biological Technology Co., Ltd. (Nanjing, China). Other chemicals and reagents were all of analytical grade.

### 3.2. Preparation of Sample Solutions

The solution of crude BSS was prepared according to a previously optimized method [38]. Firstly, crude medicinal herbs of Atractylodis Macrocephalae Rhizoma, Paeoniae Radix Alba, Citri Reticulatae Pericarpium, and Saposhnikoviae Radix were crushed into rough powders, and then mixed together as a ratio of 6:4:3:4. The mixed medicinal herbs were macerated for 1 h in 70% ethanol aqueous solution, and then decocted twice with 70% ethanol aqueous solution (1:12, *w*/*v*) using reflux extraction, 2 h for each time. Subsequently, the solution was filtered through a filter paper. The filtrates were combined together and concentrated under vacuum at 55 °C to a density of 1.6 g/mL for oral administration of rats. Similarly, the solution of processed BSS was prepared in the accordant procedure using processed Atractylodis Macrocephalae Rhizoma, processed Paeoniae Radix Alba, processed Citri Reticulatae Pericarpium, and crude Saposhnikoviae Radix.

### 3.3. Animals

Male Sprague-Dawley rats weighing 240–260 g were supplied by Vital River Laboratory Animal Technology Co., Ltd. (Beijing, China, Specific Pathogen-Free grade, certificate No.: SCXK-2016-0011). The humidity and temperature of the experimental environment were kept constant, and light/dark conditions were altered for each 12 h every day. Standard food and water were provided ad libitum. Prior to the experiments, all the rats were acclimatized to the laboratory environment for one week. Before model establishment, rats were fasted for 24 h with water taken freely. The animal experiment was conducted according to the Guide for the Care and Use of Laboratory Animals. The animal facilities and protocols were approved by the Animal Ethics Committee of Nanjing University of Chinese Medicine.

### 3.4. Induction of Ulcerative Colitis and Experimental Design

In this study, experimental ulcerative colitis was induced with TNBS enema by a published method with some modification [39]. Briefly, 24 h fasted rats were anesthetized with intraperitoneal injection of 1.5% pentobarbital sodium aqueous solution (3 mL/kg) and residual feces at the end of the rectum were gently squeezed out. Then a disposable silicone catheter with an external diameter of 2.0 mm was inserted rectally into the colon at 8 cm proximal to the anus verge by vaseline lubrication. TNBS dissolved in 30% (*v*/*v*) ethanol solution was slowly instilled into the colon via the catheter at a dose of 100 mg/kg to induce ulcerative colitis. In the sham control experiments, rats received saline alone using the same method. Rats were kept in a head-down position for 1 min after the instillation, in order to prevent leakage and distribute the agents within the entire colon.

After colon instillation, rats were randomly divided into five groups with 10 rats of each. Group 1 (sham control) and group 2 (TNBS control) were administrated with saline, group 3 (crude BSS) and group 4 (processed BSS) were given crude and processed BSS respectively at a dose of 16 g/kg, and group 5 (SASP) was given SASP at a dose of 300 mg/kg. All the treatment drugs, including saline, SASP, and crude and processed BSS, were given to rats by gavage once a day for 21 consecutive days. Blood samples (about 0.8 mL) of rats in groups 1–4 were collected from the orbital vein in centrifuge tubes at 90 min of day 3, 6, 9, 13, 17, and 21 of administration, respectively. The collected blood samples were then kept for 30 min at room temperature and centrifuged at 4500 r/min for 5 min, and the supernatant serums were stored at –80 °C for further analysis. 24 h after the last treatment, all rats were sacrificed under anesthesia. The distal 10 cm portion of colon was excised, dissected along the longitudinal mesentery, freed of adherent adipose tissue, rinsed with ice-cold saline, and stored at –80 °C for efficacy evaluation.

### 3.5. UHPLC/Q-TOF-MS/MS Analysis

#### 3.5.1. Instruments

For analysis of the multiple constituents absorbed into blood, a Shimadzu LC-30A UHPLC system (Shimadzu, Kyoto, Japan) coupled to a hybrid quadrupole time-of-flight tandem mass spectrometer (LC/MS-Triple TOF^TM^ 5600^+^, AB Sciex, Concord, ON, Canada) equipped with an electrospray ionization (ESI) interface was employed. The Analyst TF 1.6 software was installed for high efficiency data collection and processing.

#### 3.5.2. Chromatographic Separation

Chromatographic separation was performed on a Zorbax Extend C_18_ UHPLC column (2.1 mm × 100 mm, 1.8 µm, Agilent, Palo Alto, CA, USA). The column temperature was set at 25 °C. Flow rate was 0.3 mL/min and the sample injection volume was 3 μL. The mobile phase consisted of solvent A (water) and solvent B (acetonitrile). The optimized UHPLC gradient elution program was as follows: 0–20 min, 5–70% B; 20–24 min, 70–100% B; and 24–26 min, 100% B.

#### 3.5.3. Mass Spectrometry

The mass spectrometry was operated in both positive and negative ion modes. The MS method consisted of a TOF MS scan and IDA-fragmentation TOF MS/MS scan with mass ranges of *m*/*z* 100–1500 and 50–1000, respectively. The parameter settings were optimized as follows: ion spray voltage floating, +5500/–4500 V; turbo spray temperature, 550 °C; declustering potential, 60 V; nebulizer gas (gas 1), 55 psi; heater gas (gas 2), 55 psi; curtain gas, 35 psi. Nitrogen was used as the nebulizer and auxiliary gas. Collision energy (CE) of TOF MS and TOF MS/MS were set at 10 V and 40 V, respectively. Dynamic background subtraction (DBS) triggering information-dependent acquisition (IDA) MS/MS mode was used to trigger acquisition of MS/MS for constituents at low signal levels. It could combine a TOF-MS survey scan with a maximum of eight corresponding candidate MS/MS events in one experimental period simultaneously. One experimental period of 840 ms was composed of nine experiments, 200 ms of scanning time for TOF MS experiment, and 80 ms of scanning time for other eight experiments of TOF MS/MS. In addition, an automated calibration system for mass accuracy (calibrant delivery system) was equipped to carry out the recalibration every six injections.

#### 3.5.4. Preparation of Rat Serum Samples

Liquid-liquid extraction was applied to prepare the rat serum samples. Rat serum (100 μL) was spiked into a centrifuge tube and then mixed with 1000 μL of ethyl acetate by vortex mixing for 5 min. The mixture was separated by centrifugation at 12,000 r/min for 5 min, and the upper layer was then transferred to another tube and evaporated to dryness under high purity nitrogen at room temperature. The residue was reconstituted with 100 μL of methanol, vortexed for 3 min, and centrifuged at 12,000 r/min for 5 min. The final supernatant was used for UHPLC/Q-TOF-MS/MS analysis.

#### 3.5.5. Identification of Constituents Absorbed into Blood

After data acquisition, MetabolitePilot^TM^ (version 2.0, AB Sciex, Concord, ON, Canada) and PeakView^TM^ software (version 1.2, AB Sciex) were employed to search for the constituents absorbed into blood. Firstly, by searching from such databases as SciFinder, PubMed, and CNKI, the chemical compounds reported in the literature [40,41,42,43,44,45,46] belonging to Atractylodis Macrocephalae Rhizoma, Paeoniae Radix Alba, Citri Reticulatae Pericarpium, and Saposhnikoviae Radix were all summarized to establish an in-house compounds library, which includes names, molecular formulas, and chemical structures. Meanwhile, a metabolic pathway template including phase I and phase II metabolism were concluded based on the properties of chemical structures of constituents in BSS. Then, these databases of compounds and metabolite pathways were imported into MetabolitePilot^TM^ software to analyze the prototypes and metabolites in serum of each sample using the functions of generic peak finding, predicted metabolites, isotope pattern, and mass defect. Identification of peaks was based on retention time (RT) and *m*/*z* data pairs. When ions in different samples demonstrated both the same RT (tolerance of 0.1 min) and the same *m*/*z* value (tolerance of 10 ppm), they were then defined as the same ions. These procedures were completed by comparing a control sample to eliminate those chromatographic peaks belonging to endogenous components. Afterwards, PeakView^TM^ software was used to confirm the validity of the identified compounds according to mass accuracy and matching rate of MS/MS fragmentation profiles.

### 3.6. Evaluation of Anti-Ulcerative Colitis Effects

#### 3.6.1. Determination of TNF-α, IL-6, IL-10, and TGF-β1 Levels in Rat Serums

Blood samples in group 1–4 collected at different time were used to assess the cytokine levels after administration of crude and processed BSS. The levels of TNF-α, IL-6, IL-10, and TGF-β1 in rat serums were measured by enzyme-linked immunosorbent assay (ELISA) according to the manufacturer’s instruction (Jin Yibai Biological Technology Co., Ltd.). Absorbance was measured with a microplate reader at 450 nm.

#### 3.6.2. Assessment of Disease Activity Index

Disease activity index (DAI) was assessed based on weight loss, stool consistency, and blood in stool. Severity of the DAI was scored on a scale of 0–4 according to the previously scoring system [47] described by Table 5. All groups of rats were monitored after 20 days of administration to assess the disease process and treatment effect. The results of DAI were expressed as the average score of weight loss, stool consistency, and blood in stool.

#### 3.6.3. Assessment of Colon Damage by Macroscopic and Histopathological Study

Macroscopic colon damage was scored on a scale of 0–5 by the pathologists who were blinded to the group, using a previously validated scoring system with slight modifications [48]. Briefly, scoring of macroscopic colon damage was as follows: 0, no ulcer and no inflammation; 1, local hyperemia, no ulcer, and smooth colon surface; 2, hyperemia edema, slight ulcer, and ankylenteron; 3, moderate hyperemia edema, local colonic wall thickening, and mucosal necrosis and ulcer extending less than 1 cm^2^; 4, severe hyperemia edema, colonic wall thickening or necrosis (2–4 cm^2^), and mucosal necrosis and ulcer extending more than 1 cm^2^; 5, severe hyperemia edema, extensive colonic wall thickening or necrosis (>4 cm^2^), and mucosal necrosis and ulcer extending more than 1 cm^2^. After macroscopic observation, about 0.5 cm of the representative colon tissue in each rat was taken from the region of the inflamed site and was fixed in 4% paraformaldehyde for histopathological section. The fixed tissues were dehydrated, embedded in paraffin, and finally 4 μm thick sections were obtained. The samples were then deparaffinized, hydrated, cleared with xylene, and stained with hematoxylin and eosin (HE) for histological assessment.

#### 3.6.4. Western Blot Analysis

The protein concentration was determined using the BCA procedure (Thermo, Waltham, MA, USA). Protein in the colon tissue from each sample was resolved by SDS-PAGE electrophoresis, transferred to polyvinylidene fluoride (PVDF) membranes, and then blocked with 5% non-fat dry milk at room temperature for 120 min. After washing with PBS containing 0.05% Tween-20 (PBST) for three times, membranes were incubated overnight at 4 °C with TLR4, IL-1R1, MyD88, NF-κB p65, and β-tubulin antibodies. The membranes were then washed three times in PBST and incubated at room temperature for 120 min with the corresponding secondary antibody conjugated to horseradish peroxidase. The densities of protein bands were quantified by the average ratios of integral optic density following normalization to the β-tubulin expression.

#### 3.6.5. Statistical Analysis

All the results were expressed as mean ± SD. Statistical analysis was performed with SPSS 22.0 software. One way analysis of variance and Bonferroni *t* test were used to analyze the statistical significance of any difference among groups. *p*-values less than 0.05 were considered significantly different.

### 3.7. Gray Correlation Analysis

The basic idea of GCA is to investigate on the correlation degree between two sets of variations. In this work, GCA was applied to find out the spectrum-effect relationships between peak areas of diverse compounds absorbed into blood in UHPLC fingerprints after oral administration of crude and processed BSS and their corresponding biological activities in ulcerative colitis rats at different time-points during the treatment process.

In detail, set *X*_0_ = {*X*_0_(*k*)|*k* = 1, 2, ..., *n*} = (*x*_0_(1), *x*_0_(2), ..., *x*_0_(*n*)) as the sequence of system behavior characteristics. Set *X_i_* = {*X_i_*(*k*)|*k* = 1, 2, ..., *n*} = (*x_i_*(1), *x_i_*(2), ..., *x_i_*(*n*)) as the sequence of system of associated factors. Among it, *k* refers to the different time-points. 

Then, the correlation coefficient is defined as follows:

(1) $$\zeta (X_{0}(k),\;X_{i}(k))=\frac{\underset{i}{\min}\;\underset{k}{\min}\left|X_{0}(k)-X_{i}(k)\right|+\rho \underset{i}{\max}\;\underset{k}{\max}\left|X_{0}(k)-X_{i}(k)\right|}{\left|X_{0}(k)-X_{i}(k)\right|+\rho \underset{i}{\max}\;\underset{k}{\max}\left|X_{0}(k)-X_{i}(k)\right|}$$

*ρ* is the distinctive coefficient lying between 0–1, and it is generally set as 0.5.

The gray correlation degree is the arithmetic mean of the correlation coefficient at different time-points, and is formulated as follows:

(2) $$\zeta (X_{0},\;X_{i})=\frac{1}{n}\sum_{k=1}^{n}\zeta (X_{0}(k),\;X_{i}(k))$$

In this work, serum cytokine levels were selected as the pharmacodynamic indexes for GCA study. Prior to the GCA fitting, data pre-processing procedure should be made to the pharmacodynamic results due to the difference of positive and negative correlation between TNF-α, IL-6, IL-10, and TGF-β1 levels and the drug efficacy. Accordingly, the inhibition rate of TNF-α and IL-6 and the promotion rate of IL-10 and TGF-β1 were used to perform the GCA calculations instead of the absolute values of these inflammatory cytokines [49]. Specifically, the algorithm formula was as follows:

(3) $$\mathrm{Inhibition}/\mathrm{promotion}\;\mathrm{rate}=\frac{\mathrm{cytokine}\;\mathrm{levels}\;\mathrm{in}\;\mathrm{TNBS}\;\mathrm{group}-\mathrm{cytokine}\;\mathrm{levels}\;\mathrm{in}\;\mathrm{treatment}\;\mathrm{group}}{\mathrm{cytokine}\;\mathrm{levels}\;\mathrm{in}\;\mathrm{TNBS}\;\mathrm{group}-\mathrm{cytokine}\;\mathrm{levels}\;\mathrm{in}\;\mathrm{sham}\;\mathrm{control}\;\mathrm{group}}$$

Then, the inhibition and promotion rates of serum cytokine levels in crude and processed BSS groups at different time-points were set as the sequence of system behavior characteristics, while the UHPLC peak areas of diverse compounds absorbed into blood were set as sequence of system associated factors. Due to the different dimensions of two sets of data, dimensionless processing should be carried out on the raw data to eliminate the adverse effects brought by inconsistent units. Specially for dimensionless processing, each value of data was divided by the average value of the corresponding sequence, and the resultant data would be employed to calculate the correlation coefficients.

## 4. Conclusions

In this study, a GCA statistical method combining UHPLC/Q-TOF-MS/MS chemical profile and biological effect evaluation was first developed and successfully applied to investigate the underlying correlation between constituents absorbed into blood and bioactivities of crude and processed BSS. The results showed that processed BSS had better efficacy than crude BSS for the treatment of ulcerative colitis. The material basis of processing that contributed to the difference of therapeutic effects in crude and processed BSS was also found. In a word, our study can provide a scientific foundation for safety and efficacy of BSS in the clinic. Moreover, the strategy of this research could provide a valuable reference for revealing the therapeutic material basis of other TCMs or complex drug systems.

## Figures and Tables

**Table 1:** Identification of constituents absorbed into blood of crude and processed BSS based on UHPLC/Q-TOF-MS/MS.

No.	T_R_ (min)	Compound Name	Pathway	Formula	Ion Type	Found Mass	Mass Error (ppm)	MS/MS (*m*/*z*)
1	0.71	D-Mannose	parent	C_6_H_12_O_6_	[M − H]^−^	179.0559	4.9	113.0289, 75.0092, 71.0159, 59.0148
2	0.99	Narirutin-M1	loss of rutinose + ring opening	C_9_H_10_O_3_	[M − H]^−^	165.0550	2.1	119.0508, 106.0425, 72.9962, 117.0341
3	0.99	Narirutin-M2	loss of rutinose + ring opening + deethylation	C_7_H_6_O_3_	[M − H]^−^	137.0244	7.6	93.0389, 65.0463, 75.0297, 65.0131
4	1.01	Paeoniflorin-M1	loss of benzoyl	C_16_H_24_O_10_	[M − H]^−^	375.1281	−1.3	165.0556, 177.0550, 121.0307, 195.0665
5	1.01	Paeoniflorin-M2	loss of C16H22O9 + glycine conjugation	C_9_H_9_NO_3_	[M − H]^−^	178.0507	4.9	77.0416, 132.0442, 93.0352, 79.9587
6	1.05	Paeoniflorin-M3	loss of C16H22O9 + glutamine conjugation	C_12_H_14_N_2_O_4_	[M − H]^−^	249.0870	−0.1	144.0449, 131.0388, 118.0661, 128.0503
7	1.49	Dimethyl anthranilate	parent	C_9_H_11_NO_2_	[M + H]^+^	166.0860	−1.3	103.0548, 120.0813, 77.0397, 91.0547
8	1.92	Ethyl gallate-M1	dehydroxylation + sulfate conjugation	C_9_H_10_O_7_S	[M − H]^−^	261.0067	1.3	166.0269, 151.0031, 181.0494, 116.0515
9	2.14	Paeoniflorin-M4	loss of benzoyl + methylation	C_17_H_26_O_10_	[M − H]^−^	389.1440	−0.7	177.0541, 149.0600, 165.0552, 134.0367
10	2.18	Ethyl gallate-M2	sulfate conjugation	C_9_H_10_O_8_S	[M − H]^−^	277.0017	1.4	197.0444, 169.0137, 125.0242, 140.0096
11	2.76	Paeoniflorin-M5	loss of glucose and benzoyloxy + hydrogenation	C_10_H_16_O_4_	[M − H]^−^	199.0969	2.2	121.0627, 137.0987, 59.0272, 109.0680
12	3.16	Hesperidin-M1	loss of methoxyl and rhamnopyranosyl	C_21_H_22_O_10_	[M − H]^−^	433.1116	−3.0	257.0780, 113.0236, 85.0289, 109.0282
13	4.01	Synephrine-M1	*N*-acetylation	C_11_H_15_NO_3_	[M + H]^+^	210.1125	−0.1	121.0648, 149.0816, 77.0407, 91.0554
14	4.31	Tangeretin-M1	*di*-demethylation	C_18_H_16_O_7_	[M + H]^+^	345.0954	−4.3	345.1044, 128.9559, 144.9268, 176.8732
15	4.55	Atractylenolide I-M1	glucuronidation + hydrogenation	C_21_H_28_O_8_	[M + H]^+^	409.1841	−3.9	128.9538, 409.1823, 144.9273, 234.9163
16	4.74	Tangeretin-M2	*di*-demethylation	C_18_H_16_O_7_	[M + H]^+^	345.0958	−3.2	345.1003, 128.9549, 144.9214, 176.8738
17	4.75	Cimifugin-M1	hydroxylation	C_16_H_18_O_7_	[M + H]^+^	323.1135	2.8	232.0362, 247.0576, 59.0510, 323.1131
18	5.01	Albiflorin	parent	C_23_H_28_O_11_	[M + Na]^+^	503.1538	2.7	503.1514, 341.0949, 219.0608, 133.0624
19	5.30	Paeoniflorin-M6	internal hydrolysis	C_23_H_30_O_12_	[M − H]^−^	497.1632	−4.3	121.0259, 195.0692, 224.8966, 310.8794
20	5.30	Tangeretin-M3	demethylation + glucuronidation	C_25_H_26_O_13_	[M + H]^+^	535.1456	1.8	359.1125, 344.0907, 329.0667, 535.1495
21	5.31	Paeoniflorin-M7	methylation	C_24_H_30_O_11_	[M + COOH]^−^	539.1748	−2.1	121.0303, 294.9025, 165.0565, 416.8496
22	5.34	Paeoniflorin-M8	loss of hydroxymethylene	C_22_H_26_O_10_	[M − H]^−^	449.1433	−2.1	121.0300, 165.0579, 77.0410, 113.0266
23	5.36	Prim-*O*-glucosyl-cimifugin	parent	C_22_H_28_O_11_	[M + H]^+^	469.1711	1.3	261.1128, 290.1159, 307.1182, 469.1671
24	5.36	4-Methoxycinnamic acid-M1	ketone formation	C_10_H_8_O_4_	[M + H]^+^	193.0496	0.3	133.0279, 178.0267, 122.0359, 150.0318
25	5.38	Paeoniflorin	parent	C_23_H_28_O_11_	[M − H]^−^	479.1534	−2.8	179.0702, 151.0744, 133.0641, 449.1466
26	5.38	sec-*O*-Glucosyl-hamaudol-M1	phosphorylation	C_21_H_27_O_13_P	[M + H]^+^	519.1264	0.3	201.0063, 519.1267, 235.0274, 323.0431
27	5.40	Atractylenolide III-M1	hydroxylation + internal hydrolysis	C_15_H_22_O_5_	[M + H]^+^	283.1548	2.9	131.0834, 201.1246, 143.0798, 157.1025
28	5.40	Atractylenolide II-M1	di-hydroxylation + internal hydrolysis	C_15_H_22_O_5_	[M + H]^+^	283.1548	2.9	131.0834, 201.1246, 143.0798, 157.1025
29	5.49	Tangeretin-M4	*di*-demethylation	C_18_H_16_O_7_	[M + H]^+^	345.0956	−3.9	345.0958, 110.9771, 345.1186, 91.0523
30	5.49	Cimifugin-M2	hydroxylation	C_16_H1_8_O_7_	[M + H]^+^	323.1135	3.0	232.0369, 247.0607, 59.0514, 203.0337
31	5.64	Nobiletin-M1	demethylation + glucuronidation	C_26_H_28_O_14_	[M + H]^+^	565.1559	1.3	389.1243, 359.0826, 565.1626, 124.0898
32	5.72	Dimethyl anthranilate-M1	methylation	C_10_H_13_NO_2_	[M + H]^+^	180.1019	−0.1	56.9678, 120.0807, 162.0895, 69.0347
33	5.84	3,5,6,7,8,3′,4′-Hepta-methoxyflavone-M1	demethylation + glucuronidation	C_27_H_30_O_15_	[M + H]^+^	595.1671	2.3	419.1340, 389.0892, 404.1141, 595.1714
34	5.85	Atractylenolide I-M2	hydroxylation + internal hydrolysis	C_15_H_20_O_4_	[M + H]^+^	265.1434	2.5	153.0705, 128.0599, 131.0845, 159.0790
35	5.99	Nobiletin-M2	demethylation + glucuronidation	C_26_H_28_O_14_	[M + H]^+^	565.1558	1.1	389.1251, 359.0829, 374.1053, 565.1465
36	6.10	Nodakenetin-M1	hydroxylation	C_14_H_14_O_5_	[M + H]^+^	263.0916	0.8	203.0707, 245.0771, 175.0378, 128.0632
37	6.14	Atractylenolide II-M2	di-hydroxylation	C_15_H_20_O_4_	[M + H]^+^	265.1439	1.7	143.0855, 91.0562, 117.0658, 122.0720
38	6.20	Scopoletin	parent	C_10_H_8_O_4_	[M + H]^+^	193.0496	0.2	178.0260, 150.0313, 133.0292, 94.0423
39	6.35	Albiflorin-M1	loss of glucose and benzoyloxy	C_10_H_14_O_3_	[M + H]^+^	183.1014	−0.9	56.9661, 109.0640, 119.0856, 91.0536
40	6.43	Prim-*O*-glucosyl-cimifugin-M1	loss of glucose	C_16_H_18_O_6_	[M + H]^+^	307.1185	3.0	259.0611, 235.0609, 221.0453, 307.1191
41	6.43	Cimifugin	parent	C_16_H_18_O_6_	[M + H]^+^	307.1185	3.0	259.0611, 235.0609, 221.0453, 307.1191
42	6.44	Tangeretin-M5	loss of methoxyl + demethylation	C_18_H_16_O_6_	[M + H]^+^	329.1005	−4.6	329.1011, 314.0763, 98.9837, 255.0262
43	6.44	Tetramethyl-*O*-scutellarin-M1	demethylation	C_18_H_16_O_6_	[M + H]^+^	329.1005	−4.6	329.1011, 314.0763, 98.9837, 255.0262
44	6.66	5-*O*-Methyl-visamminol-M1	hydroxylation	C_16_H_18_O_6_	[M + H]^+^	307.1188	4.0	259.0603, 235.0603, 307.1185, 221.0447
45	6.66	4′-*O*-β-D-Glucosyl-5-*O*-methylvisamminol-M1	loss of glucose + hydroxylation	C_16_H_18_O_6_	[M + H]^+^	307.1188	4.0	259.0603, 235.0603, 307.1185, 221.0447
46	6.74	Prim-*O*-glucosyl-cimifugin-M2	dehydroxylation	C_22_H_28_O_10_	[M + H]^+^	453.1764	1.9	291.1236, 273.1130, 231.0647, 453.1768
47	6.74	4′-*O*-β-D-Glucosyl-5-*O*-methyl-visamminol	parent	C_22_H_28_O_10_	[M + H]^+^	453.1764	1.9	291.1236, 273.1130, 231.0647, 453.1768
48	6.78	Atractylenolide III-M2	internal hydrolysis	C_15_H_22_O_4_	[M + H]^+^	267.1597	2.2	131.0848, 157.1008, 185.1323, 143.0858
49	6.78	Atractylenolide II-M3	hydroxylation + internal hydrolysis	C_15_H_22_O_4_	[M + H]^+^	267.1597	2.2	131.0848, 157.1008, 185.1323, 143.0858
50	6.78	Albiflorin-M2	loss of glucose and benzoyloxy + methylation	C_11_H_16_O_3_	[M + H]^+^	197.1170	−0.9	91.0542, 105.0681, 105.0768, 114.9465
51	6.87	Atractylenolide III-M3	hydroxylation	C_15_H_20_O_4_	[M + H]^+^	265.1437	1.1	143.0843, 119.0928, 105.0698, 201.1283
52	6.87	Atractylenolide II-M4	dihydroxylation	C_15_H_20_O_4_	[M + H]^+^	265.1437	1.1	143.0843, 119.0928, 105.0698, 201.1283
53	6.97	Hesperidin-M2	loss of rutinose + internal hydrolysis	C_16_H_16_O_6_	[M + H]^+^	305.1026	2.2	275.0558, 233.0442, 305.0996, 247.0611
54	7.02	Nodakenetin-M2	hydroxylation	C_14_H_14_O_5_	[M + H]^+^	263.0921	2.7	191.0328, 203.0700, 217.0873, 91.0550
55	7.14	5-*O*-methyl-visamminol-M2	hydroxylation	C_16_H_18_O_6_	[M + H]^+^	307.1191	4.8	259.0610, 307.1203, 136.9283, 221.0436
56	7.14	4′-*O*-β-D-glucosyl-5-*O*-methylvisamminol-M2	loss of glucose + hydroxylation	C_16_H_18_O_6_	[M + H]^+^	307.1191	4.8	259.0610, 307.1203, 136.9283, 221.0436
57	7.15	Hesperidin	parent	C_28_H_34_O_15_	[M − H]^−^	609.1792	−3.7	301.0707, 609.1770, 325.0707, 242.0497
58	7.63	Atractylenolide III-M4	hydroxylation	C_15_H_20_O_4_	[M + H]^+^	265.1439	1.7	91.0561, 128.0621, 141.0754, 155.0899
59	7.63	Atractylenolide II-M5	di-hydroxylation	C_15_H_20_O_4_	[M + H]^+^	265.1439	1.7	91.0561, 128.0621, 141.0754, 155.0899
60	7.93	Scopoletin-M1	methylation	C_11_H_10_O_4_	[M + H]^+^	207.0654	1.2	191.0331, 151.0734, 163.0391, 107.0499
61	7.93	Scoparone	parent	C_11_H_10_O_4_	[M + H]^+^	207.0654	1.2	191.0331, 151.0734, 163.0391, 107.0499
62	8.03	Cimifugin-M3	loss of methoxyl + dehydroxylation	C_15_H_16_O_4_	[M + H]^+^	261.1129	2.7	131.0501, 189.0505, 77.0424, 128.8958
63	8.04	5-*O*-Methyl-visamminol-M3	demethylation and hydroxylation	C_15_H_16_O_6_	[M + H]^+^	293.1026	2.1	221.0443, 205.0473, 293.1043, 233.0462
64	8.45	Hesperidin-M3	loss of rutinose + deme-thylation + di-hydrogenation	C_15_H_16_O_6_	[M − H]^−^	291.0863	−0.1	273.0748, 233.0443, 247.1694, 175.0364
65	8.52	Nodakenetin	parent	C_14_H_14_O_4_	[M + H]^+^	247.0972	3.0	247.0958, 229.0855, 175.0374, 147.0439
66	8.52	5-*O*-Methyl-visamminol	parent	C_16_H_18_O_5_	[M + H]^+^	291.1238	3.7	243.0657, 219.0650, 217.0504, 205.0488
67	8.52	4′-*O*-β-D-glucosyl-5-*O*-methyl-visamminol-M3	loss of glucose	C_16_H_18_O_5_	[M + H]^+^	291.1238	3.7	243.0657, 219.0650, 217.0504, 205.0488
68	8.85	sec-*O*-Glucosyl-hamaudol	parent	C_21_H_26_O_10_	[M + H]^+^	439.1599	−0.1	277.1064, 259.0971, 217.0497, 205.0498
69	8.96	Nodakenetin-M3	isomerization	C_14_H_14_O_4_	[M + H]^+^	247.0969	1.7	213.0561, 175.0361, 229.0861, 171.0444
70	9.08	3,5,7,3′,4′-Penta-methoxyflavone-M1	di-demethylation	C_18_H_16_O_7_	[M + H]^+^	345.0987	5.1	297.0465, 315.0535, 98.9818, 130.9360
71	9.21	Sinensetin-M1	demethylation	C_19_H_18_O_7_	[M + H]^+^	359.1133	2.0	329.0663, 359.1366, 301.0538, 163.0718
72	9.35	Nobiletin-M3	*di*-demethylation	C_19_H_18_O_8_	[M + H]^+^	375.1078	1.0	345.0597, 197.0133, 327.0633, 375.1007
73	9.36	Nobiletin-M4	loss of methoxyl and methoxyl + demethylation	C_18_H_16_O_6_	[M + H]^+^	329.1030	3.1	299.0562, 329.1027, 271.0589, 153.0170
74	9.36	Sinensetin-M2	loss of methoxyl + demethylation	C_18_H_16_O_6_	[M + H]^+^	329.1030	3.1	299.0562, 329.1027, 271.0589, 153.0170
75	9.36	Tangeretin-M6	loss of methoxyl + demethylation	C_18_H_16_O_6_	[M + H]^+^	329.1030	3.1	299.0562, 329.1027, 271.0589, 153.0170
76	9.36	Tetramethyl-*O*-scutellarin-M2	demethylation	C_18_H_16_O_6_	[M + H]^+^	329.1030	3.1	299.0562, 329.1027, 271.0589, 153.0170
77	9.61	Nobiletin-M5	loss of methoxyl + demethylation	C_19_H_18_O_7_	[M + H]^+^	359.1137	3.3	329.0647, 359.1146, 314.0405, 149.0582
78	9.61	Tangeretin-M7	demethylation	C_19_H_18_O_7_	[M + H]^+^	359.1137	3.3	329.0647, 359.1146, 314.0405, 149.0582
79	9.79	Nobiletin-M6	demethylation	C_20_H_20_O_8_	[M + H]^+^	389.1238	1.8	359.0756, 389.1259, 313.0711, 301.1398
80	9.85	Atractylenolide I-M3	internal hydrolysis	C_15_H_20_O_3_	[M + H]^+^	249.1489	1.7	142.0767, 128.0643, 157.1014, 185.1333
81	10.03	3,5,7,3′,4′-Penta-methoxyflavone-M2	demethylation	C_19_H_18_O_7_	[M + H]^+^	359.1136	2.8	326.0808, 298.0865, 162.0665, 344.0917
82	10.15	Nobiletin-M7	loss of methoxyl and methoxyl + demethylation	C_18_H_16_O_6_	[M + H]^+^	329.1025	1.7	299.0528, 271.0596, 329.1016, 314.0764
83	10.15	Sinensetin-M3	loss of methoxyl + demethylation	C_18_H_16_O_6_	[M + H]^+^	329.1025	1.7	299.0528, 271.0596, 329.1016, 314.0764
84	10.15	Tangeretin-M8	loss of methoxyl + demethylation	C_18_H_16_O_6_	[M + H]^+^	329.1025	1.7	299.0528, 271.0596, 329.1016, 314.0764
85	10.15	Tetramethyl-*O*-scutellarin-M3	demethylation	C_18_H_16_O_6_	[M + H]^+^	329.1025	1.7	299.0528, 271.0596, 329.1016, 314.0764
86	10.25	Nobiletin-M8	demethylation	C_20_H_20_O_8_	[M + H]^+^	389.1238	1.7	359.0764, 331.0804, 389.1241, 356.0851
87	10.25	Tangeretin-M9	hydroxylation	C_20_H_20_O_8_	[M + H]^+^	389.1238	1.7	359.0764, 331.0804, 389.1241, 356.0851
88	10.64	Xanthotoxin-M1	hydrogenation	C_12_H_10_O_4_	[M + H]^+^	219.0655	1.4	203.0339, 204.0417, 147.0448, 115.0538
89	10.71	Atractylenolide I-M4	hydroxylation + hydrogenation	C_15_H_20_O_3_	[M + H]^+^	249.1485	−0.2	119.0870, 185.1335, 109.0634, 128.0597
90	10.94	Xanthotoxin	parent	C_12_H_8_O_4_	[M + H]^+^	217.0493	−1.1	202.0254, 174.0301, 161.0594, 118.0428
91	10.96	3,5,6,7,8,3′,4′-Hepta-methoxyflavone-M2	demethylation	C_21_H_22_O_9_	[M + H]^+^	419.1346	2.3	389.0868, 419.1337 371.0762, 404.1100
92	11.03	Nobiletin-M9	demethylation	C_20_H_20_O_8_	[M + H]^+^	389.1232	0.3	359.0775, 341.0667, 389.1229, 374.1010
93	11.03	Sinensetin-M4	hydroxylation	C_20_H_20_O_8_	[M + H]^+^	389.1232	0.3	359.0775, 341.0667, 389.1229, 374.1010
94	11.03	3,5,6,7,8,3′,4′-Hepta-methoxyflavone-M3	loss of methoxyl + demethylation	C_20_H_20_O_8_	[M + H]^+^	389.1232	0.3	359.0775, 341.0667, 389.1229, 374.1010
95	11.19	Sinensetin	parent	C_20_H_20_O_7_	[M + H]^+^	373.1293	3.1	373.1293, 358.1059, 343.0841, 181.0142
96	11.21	Nobiletin-M10	demethylation	C_20_H_20_O_8_	[M + H]^+^	389.1238	1.8	359.0775, 341.0667, 389.1229, 374.1010
97	11.30	Nobiletin-M11	loss of methoxyl + demethylation	C_19_H_18_O_7_	[M + H]^+^	359.1138	3.5	329.0674, 283.0608, 359.1128, 311.0561
98	11.30	Tangeretin-M10	demethylation	C_19_H_18_O_7_	[M + H]^+^	359.1138	3.5	329.0674, 283.0608, 359.1128, 311.0561
99	11.30	3,5,6,7,8,3′,4′-Hepta-methoxyflavone-M4	loss of methoxyl and methoxyl + demethylation	C_19_H_18_O_7_	[M + H]^+^	359.1138	3.5	329.0674, 283.0608, 359.1128, 311.0561
100	11.32	3,5,6,7,8,3′,4′-Hepta-methoxyflavone-M5	demethylation	C_21_H_22_O_9_	[M + H]^+^	419.1340	0.9	389.0873, 419.1316, 371.0760, 403.1038
101	11.56	Nobiletin-M12	demethylation	C_20_H_20_O_8_	[M + H]^+^	389.1241	2.7	359.0777, 389.1247, 374.1014, 344.0542
102	11.56	Tangeretin-M11	hydroxylation	C_20_H_20_O_8_	[M + H]^+^	389.1241	2.7	359.0777, 389.1247, 374.1014, 344.0542
103	11.56	3,5,6,7,8,3′,4′-Hepta-methoxyflavone-M6	loss of methoxyl + demethylation	C_20_H_20_O_8_	[M + H]^+^	389.1241	2.7	359.0777, 389.1247, 374.1014, 344.0542
104	11.58	Nobiletin-M13	hydroxylation	C_21_H_22_O_9_	[M + H]^+^	419.1340	0.9	389.0882, 419.1355, 371.0750, 404.1151
105	11.58	3,5,6,7,8,3′,4′-Hepta-methoxyflavone-M7	demethylation	C_21_H_22_O_9_	[M+H]^+^	419.1340	0.9	389.0882, 419.1355, 371.0750, 404.1151
106	11.81	Hesperidin-M4	dehydroxylation + loss of rhamnopyranose and hydroxymethylene	C_21_H_22_O_8_	[M + H]^+^	403.1397	2.3	373.0922, 403.1395, 327.0848, 342.1096
107	11.87	Atractylenolide II-M6	hydroxylation	C_15_H_20_O_3_	[M + H]^+^	249.1491	2.2	131.0866, 142.0778, 157.1045, 117.0668
108	11.89	sec-*O*-Glucosyl-hamaudol-M2	loss of glucose	C_15_H_16_O_5_	[M + H]^+^	277.1078	2.7	205.0492, 259.0972, 277.1067, 177.0539
109	11.89	Hamaudol	parent	C_15_H_16_O_5_	[M + H]^+^	277.1078	2.7	205.0492, 259.0972, 277.1067, 177.0539
110	11.89	3′-*O*-Angeloyl-hamaudol-M1	loss of angeloyl	C_15_H_16_O_5_	[M + H]^+^	277.1078	2.7	205.0492, 259.0972, 277.1067, 177.0539
111	11.89	3′-*O*-Acetyl-hamaudol-M1	deacetylation	C_15_H_16_O_5_	[M + H]^+^	277.1078	2.7	205.0492, 259.0972, 277.1067, 177.0539
112	12.19	Narirutin-M3	loss of rhamnopyranose	C_21_H_22_O_9_	[M + H]^+^	419.1346	2.1	389.0873, 419.1336, 404.1104, 371.0772
113	12.36	Nobiletin-M14	loss of methoxyl and methoxyl	C_19_H_18_O_6_	[M + H]^+^	343.1182	1.8	313.0700, 181.0148, 285.0767, 343.1192
114	12.36	Sinensetin-M5	loss of methoxyl	C_19_H_18_O_6_	[M + H]^+^	343.1182	1.8	313.0700, 181.0148, 285.0767, 343.1192
115	12.36	Tetramethyl-*O*-scutellarin	parent	C_19_H_18_O_6_	[M + H]^+^	343.1182	1.8	313.0700, 181.0148, 285.0767, 343.1192
116	12.68	Atractylenolide I-M5	hydroxylation	C_15_H_18_O_3_	[M + H]^+^	247.1330	0.5	153.0664, 183.1194, 56.9667, 81.0702
117	13.22	Tetramethyl-*O*-scutellarin-M4	hydroxylation + methylation	C_20_H_20_O_7_	[M+H]^+^	373.1290	2.3	343.0819, 297.0812, 312.1013, 373.1312
118	13.37	Nobiletin	parent	C_21_H_22_O_8_	[M + H]^+^	403.1397	2.3	373.0926, 403.1388, 388.1167, 358.0683
119	13.37	3,5,6,7,8,3′,4′-Hepta-methoxyflavone-M8	loss of methoxyl	C_21_H_22_O_8_	[M + H]^+^	403.1397	2.3	373.0926, 403.1388, 388.1167, 358.0683
120	13.50	Atractylenolide III-M5	hydrogenation	C_15_H_22_O_3_	[M + H]^+^	251.1640	−0.7	121.1001, 93.0710, 131.0860, 149.0220
121	13.51	3,5,6,7,8,3′,4′-Hepta-methoxyflavone-M9	demethylation	C_21_H_22_O_9_	[M + H]^+^	419.1338	0.4	226.0487, 419.1274, 178.0606, 183.0312
122	13.52	Atractylenolide I-M6	hydrogenation	C_15_H_20_O_2_	[M + H]^+^	233.1536	0.1	79.0557, 131.0837, 93.0706, 105.0720
123	14.14	3,5,6,7,8,3′,4′-Heptamethoxyflavone	parent	C_22_H_24_O_9_	[M + H]^+^	433.1503	2.4	403.1034, 433.1499, 418.1256, 385.0923
124	14.14	3,5,7,8,3′,4′-Hexa-methoxyflavone-M1	hydroxylation + methylation	C_22_H_24_O_9_	[M + H]^+^	433.1503	2.4	403.1034, 433.1499, 418.1256, 385.0923
125	14.69	Nobiletin-M15	loss of methoxyl	C_20_H_20_O_7_	[M + H]^+^	373.1292	2.6	343.0819, 373.1303, 358.1058, 211.0243
126	14.69	Tangeretin	parent	C_20_H_20_O_7_	[M + H]^+^	373.1292	2.6	343.0819, 373.1303, 358.1058, 211.0243
127	14.69	3,5,6,7,8,3′,4′-Hepta-methoxyflavone-M10	loss of methoxyl and methoxyl	C_20_H_20_O_7_	[M+H]^+^	373.1292	2.6	343.0819, 373.1303, 358.1058, 211.0243
128	14.71	3β-Hydroxy-atractylone-M1	desaturation	C_15_H_18_O_2_	[M + H]^+^	231.1383	1.3	91.0534, 128.0616, 105.0706, 143.0840
129	14.73	Atractylenolide III	parent	C_15_H_20_O_3_	[M − H]^−^	247.1332	1.1	203.1438, 187.1119, 172.0872, 147.0811
130	14.73	Atractylenolide II-M7	hydroxylation	C_15_H_20_O_3_	[M − H]^−^	247.1332	1.1	203.1438, 187.1119, 172.0872, 147.0811
131	16.59	7-OH-3,5,6,8,3′,4′-Hexamethoxy-flavone	parent	C_21_H_22_O_9_	[M + H]^+^	419.1339	0.6	419.1346, 404.1085, 389.0879, 371.0763
132	17.55	Atractylenolide I-M7	hydrogenation	C_15_H_20_O_2_	[M + H]^+^	233.1537	0.5	159.0813, 131.0867, 105.0711, 91.0555
133	17.55	Atractylenolide II	parent	C_15_H_20_O_2_	[M + H]^+^	233.1537	0.5	159.0813, 131.0867, 105.0711, 91.0555
134	19.60	Atractylenolide I	parent	C_15_H_18_O_2_	[M + H]^+^	231.1381	0.7	231.1392, 185.1334, 143.0849, 128.0633

**Table 2:** Results of body weight, DAI, and macroscopic damage scores in rats (*n* = 10).

Group	Weight/g	DAI	Macroscopic Damage Score
Sham control	390.4 ± 16.1	0.00 ± 0.00	0.00 ± 0.00
TNBS control	295.2 ± 18.0 ***	3.50 ± 0.57***	3.80 ± 0.63 ***
Crude BSS	336.6 ± 17.9 ^###^	2.27 ± 0.38 ^ΔΔΔ^	2.30 ± 0.68 ^###^
Processed BSS	371.1 ± 17.3 ^###^^ΔΔΔ^	1.33 ± 0.61 ^###^^ΔΔΔ^	1.50 ± 0.53 ^###^^ΔΔ^
SASP	374.2 ± 21.2 ^###^	1.13 ± 0.55 ^###^	1.30 ± 0.48 ^###^

Note: *** *p* < 0.001 vs. sham control; ^###^ *p* < 0.001 vs. TNBS control; ^ΔΔ^ *p* < 0.01, ^ΔΔΔ^ *p* < 0.001 vs. crude BSS.

**Table 3:** Inhibition/promotion rates of TNF-α, IL-6, IL-10, and TGF-β1 levels after consecutive administration of crude and processed BSS on day 3, 6, 9, 13, 17, and 21.

Cytokine	Crude BSS Group						Processed BSS Group					
	3 d	6 d	9 d	13 d	17 d	21 d	3 d	6 d	9 d	13 d	17 d	21 d
IL-6	0.369	0.450	0.140	0.277	0.439	0.579	0.512	0.497	0.284	0.436	0.666	0.876
IL-10	0.419	0.191	0.092	0.384	0.706	0.888	0.453	0.331	0.541	1.043	1.564	2.036
TGF-β1	0.187	0.233	0.443	0.683	0.941	1.246	0.307	0.487	0.809	1.171	1.677	2.292
TNF-α	0.195	0.222	0.303	0.443	0.569	0.674	0.298	0.377	0.511	0.659	0.822	0.928

**Table 4:** Gray correlation grades with rank order of top 10 compounds absorbed into blood.

Rank Order	Gray Correlation Grade							
	IL-6		IL-10		TGF-β1		TNF-α	
	Compound	Correlation	Compound	Correlation	Compound	Correlation	Compound	Correlation
1	Atractylenolide I-M5	0.9665	Nobiletin-M3	0.9170	3,5,6,7,8,3′,4′-Hepta-methoxyflavone-M9	0.9312	Atractylenolide I-M5	0.9339
2	Hesperidin-M2	0.9539	3,5,6,7,8,3′,4′-Hepta-methoxyflavone-M9	0.9129	Nobiletin-M3	0.9135	3,5,6,7,8,3′,4′-Hepta-methoxyflavone-M9	0.9301
3	Cimifugin	0.9534	Sinensetin-M1	0.9019	Dimethyl anthranilate-M1	0.9042	Tangeretin-M4	0.9286
4	Albiflorin-M2	0.9525	Atractylenolide I-M5	0.8962	Prim-*O*-glucosyl-cimifugin	0.9026	D-Mannose	0.9191
5	Cimifugin-M3	0.9505	Cimifugin-M1	0.8864	Atractylenolide I-M5	0.9004	Nobiletin-M3	0.9173
6	5-*O*-Methylvisamminol-M2	0.9491	Atractylenolide III-M3	0.8859	Tangeretin-M4	0.8998	Albiflorin-M1	0.9165
7	sec-*O*-Glucosyl-hamaudol	0.9443	3,5,7,3′,4′-Penta-methoxyflavone-M2	0.8813	Albiflorin-M1	0.8971	Cimifugin-M3	0.9151
8	Atractylenolide II	0.9422	Dimethyl anthranilate-M1	0.8799	Cimifugin-M1	0.8960	Atractylenolide II	0.9133
9	Atractylenolide III-M5	0.9353	Atractylenolide III	0.8781	Atractylenolide II-M2	0.8930	Nobiletin-M1	0.9086
10	Atractylenolide III-M4	0.9343	Tangeretin-M4	0.8756	Atractylenolide I-M2	0.8918	Dimethyl anthranilate-M1	0.9084

**Table 5:** Scoring criteria of disease activity index (DAI).

Score	Weight Loss (%)	Stool Consistency	Blood in Stool
0	(−)	Normal	Hemoccult (−)
1	1–5		
2	5–10	Loose	Hemoccult (+)
3	10–15		
4	>15	Diarrhoea	Gross bleeding

## Supplementary Materials

The following are available online, Figure S1: Typical total ion chromatograms (TICs) of constituents absorbed into blood of crude and processed BSS in both positive and negative ion modes. (**A**): crude BSS; (**B**): processed BSS.
