# Supplementary material for: Investigation on Spectrum-Effect Correlation between Constituents Absorbed into Blood and Bioactivities of Baizhu Shaoyao San before and after Processing on Ulcerative Colitis Rats by UHPLC/Q-TOF-MS/MS Coupled with Gray Correlation Analysis
Source: Molecules. 2019 Mar 7;24(5):940. doi: 10.3390/molecules24050940 (PMC6429276; doi:10.3390/molecules24050940)
Supplement: Supplementary file 1 [file molecules-24-00940-s001.pdf]

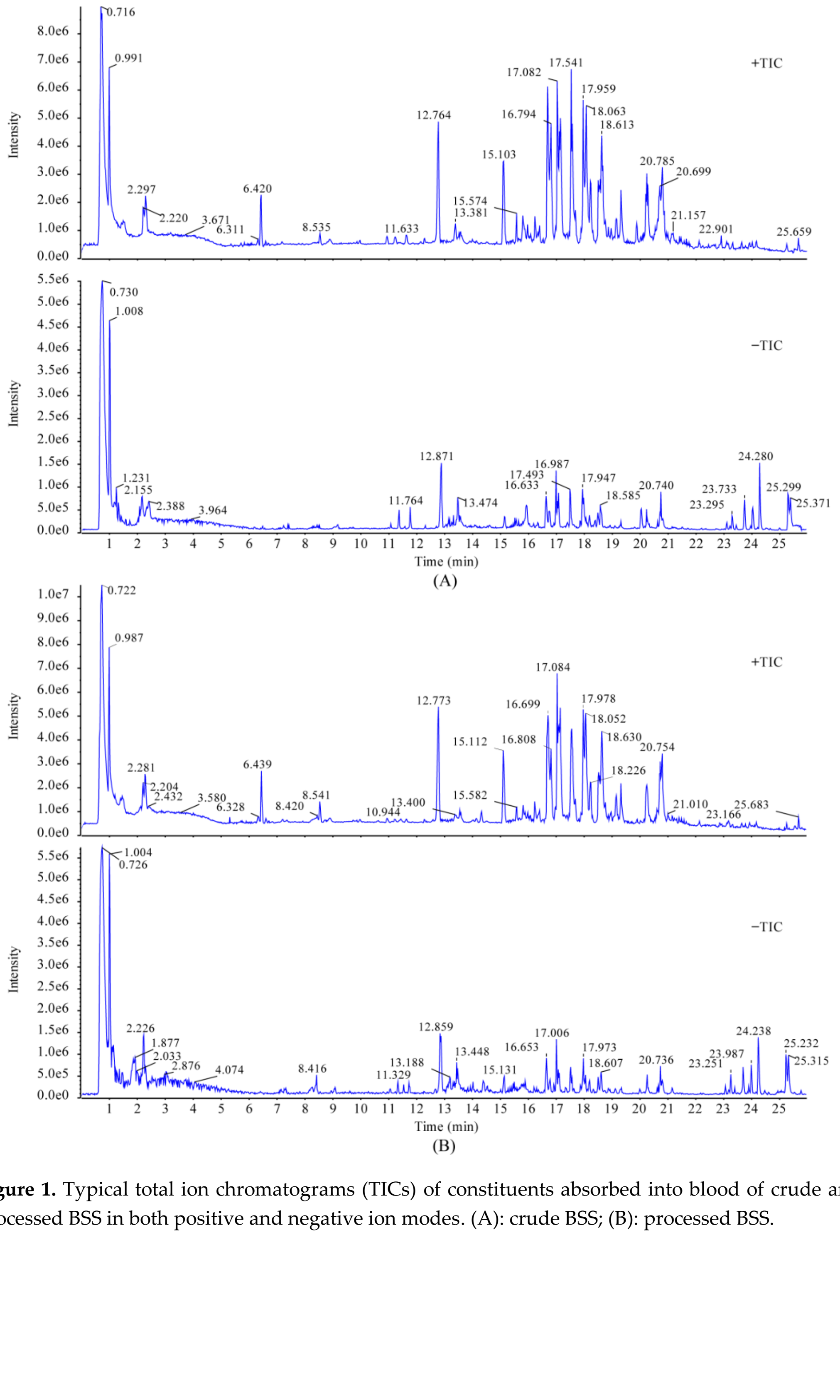

Figure 1. Typical total ion chromatograms (TICs) of constituents absorbed into blood of crude and processed BSS in both positive and negative ion modes. (A): crude BSS; (B): processed BSS.
